# Nursing's Long History of Mutual Aid

**DOI:** 10.1111/nup.70127

**Published:** 2026-10-20

**Authors:** Sam Porter

**Affiliations:** ^1^ School of Health and Care Bournemouth University Bournemouth UK

**Keywords:** anarchism, evolution, feminism, Mutual aid, nursing history

## Abstract

The doctrine of mutual aid and its application to nursing has recently enjoyed considerable attention in the pages of *Nursing Philosophy*. However, this is not the first time that nursing theorists have identified its salience to nursing. In 1907, Adelaide Nutting and Lavinia Dock specified mutual aid as the evolutionary impulse upon which the activity of nursing is founded. Nutting and Dock adopted mutual aid from Peter Kropotkin's application of Russian evolutionary theory to anarchism. Russian natural scientists argued that Darwin had overemphasised struggle and under‐emphasised cooperation in his theory of natural selection. Just as Kropotkin sought to uncover the mundane relations of mutual support that were the bedrock of species' success, Nutting and Dock aimed to bring to light the unsung history of nursing's crucial contribution to humanity through its enactment of mutual aid in healthcare. They took a gendered approach, which assumed that the ability to engage in mutual aid was a function of the capacity to care which, in turn, was more developed in women. They also took a reformist approach in contrast to their anarchist contemporaries, who advocated revolution. After addressing contemporary dilemmas about the purpose and possibility of mutual aid, the articulation of Nutting and Dock's feminism with difference and neo‐Darwinian feminisms are noted. In examining the relationship of their approach with contemporary ecological theory, Nutting and Dock's evolutionary grounding for mutual aid is compared favourably with contemporary anarchism's exclusively political approach, though both are excessively human‐centred. It is concluded that the most important message we can take from Nutting and Dock is that survival depends on mutual aid and that nurses have the potential to be key actors in its instantiation.

The modern nurse, keenly interested as she is in the present and the future of her profession, knows little of its past. She loses both the inspiration which arises from cherished tradition, and the perspective which shows the relation of one progressive movement to others. Only in the light of history can she clearly see how closely her own calling is linked with the general conditions of education and of liberty that obtain – as they rise, she rises, and as they sink, she falls.

Adelaide Nutting and Lavinia Dock (1907a, p.v)

Though we can understand imagination as speculative, a prophecy for another present/future, to do justice in the present/future, it is necessary to reflect clearly on our histories.

Jessica Dillard‐Wright (2022, p.5)

## 1 Introduction

In recent years, a number of papers addressing the potential of political anarchism have been published in *Nursing Philosophy* (Dillard‐Wright 2022; Martin and Laurin 2023; Oppedisano and Dillard‐Wright 2024; Malfait 2025; Essex 2026). Within this literature, probably the most significant conceptual tool that has emerged is the notion of mutual aid, which has its origins in the Russian anarchist, Peter Kropotkin's (1902) eponymous work that posited cooperation as the most important evolutionary imperative.

The recent interest in mutual aid has largely been generated by Dean Spade's (2020) monograph on the subject (*vide* Dillard‐Wright 2022; Martin and Laurin 2023), which in turn was stimulated by the social conditions laid bare by the Covid pandemic. However, this is not the first time that mutual aid has been a focus of nursing theorists. Remarkable as it may seem, 120 years ago it was a core concept for two of the founding mothers of American nursing theory, Mary Adelaide Nutting (1858–1948) and Lavinia Lloyd Dock (1858–1956). The purposes of this paper are to explore the intellectual and socio‐political context in which these authors developed their ideas; to explicate their interpretation of mutual aid and its application to nursing; and to tentatively explore articulations between their position and that of contemporary commentators. This articulation is important because, as Dillard‐Wright (2023) has observed, challenging established historical nursing narratives helps provide space for the development of future possibilities.

The significance of Nutting and Dock in the formation of nursing thought and organisation can be quickly gleaned from their biographies. Adelaide Nutting began her nursing career in 1889, becoming principal of the Johns Hopkins School of Nursing in 1894. She and Lavinia Dock helped found the *American Journal of Nursing* in 1900. In 1907 she was appointed to a Chair at Columbia University's Teachers' College, the first nurse ever to hold a full professorship. Nutting was also involved in the suffragette movement (Steedman 2011). Lavinia Dock enrolled to train as a nurse at the Bellevue Hospital School of Nursing in 1884. In 1893, with Nutting and Elizabeth Hampton Robb, she founded what was to become the National League for Nursing, and in 1899, in collaboration with Ethel Gordon Fenwick, she founded the International Council of Nurses. She left academia to live and work in the Henry Street Nurses' Settlement in New York City where she was a visiting nurse among immigrant communities. While at the settlement, she became highly politicised as a socialist, suffragette and pacifi*s*t (Garofalo and Fee 2015).

Together, Nutting and Dock wrote the first two volumes of 'A History of Nursing: the evolution of nursing systems from the earliest times to the foundation of the first English and American training schools for nurses' (Nutting and Dock 1907a, 1907b). Dock went on to write and edit another two volumes (Dock 1912a, 1912b) that concentrated on recent global developments in nursing, and then, with Isabel Stewart, a single volume edition for student use (Dock and Stewart 1920). The subtitle of Volumes 1 and 2 indicates both the centrality of an evolutionary approach and, in the term ‘from the earliest times’, a scope that stretches back as far as pre‐human modes of health care. In other words, their evolutionary approach is both natural and social.

In their introduction to the first volume, Nutting and Dock identify three aspects of evolution that feed into the ethos of nursing care, the first two of which were maternal care of the newborn and parental care of human infants. However, they asserted that:

There is, finally, ages back in the history of our primal ancestry, still another impulse, even more widespread and lasting, and far less personal or individualistic than either of those already mentioned; namely, the social instinct or feeling which is clearly the rudiment of what we call altruism or humanitarianism in the human race. This is the instinct of mutual aid, actually a sense of race preservation, which prompts the protection of the younger and weaker of the herd by the older and stronger, and causes flocks to share the distress or attempt the rescue of the individual in peril.

Nutting and Dock 1907a

Nutting and Dock were not shy about advertising the source of their insights: ‘To Kropotkin, whose scientific knowledge is equalled by his benevolence toward all created beings, we owe the elucidation of this truth, which he expounds most fascinatingly in his *Mutual Aid*’ (1907a).

## 2 The Intellectual Context of Mutual Aid

Before addressing the application of mutual aid to the evolution of nursing, it is useful to locate Kropotkin's theory within the wider theory of evolution, both natural and social.

### 2.1 Darwin's Theory of Evolution

Charles Darwin's theory of evolution identified what he termed ‘the struggle for existence’ (Darwin 1994) as the basis for natural selection. In conceptualising this struggle, Darwin relied heavily on Malthus's (2004) observation that populations tend to increase at a geometric rate, while available resources increase arithmetically, which leads to a struggle for survival between organisms. In that struggle, those individuals whose characteristics are best fitted to their environment will tend to survive long enough to breed, and thereby pass on their favourable characteristics. Thus, natural selection entails the survival of the fittest in ‘the great battle of life’ (Darwin 1994, p.59).

The martial imagery of ‘the great battle’ implies that the struggle for existence is characterised by brutal competition. However, in his formal definition, Darwin is more circumspect:

I should premise that I use this term in a large and metaphorical sense *including dependence of one being on another*… Two canine animals, in a time of dearth, may be truly said to struggle with each other which shall get food and live. But a plant on the edge of a desert is said to struggle for life against the drought … In these several senses, which pass into each other, I use of convenience' sake the general term of Struggle for Existence.

Darwin 1994, p.50, my emphasis

### 2.2 The Theory of Mutual Aid

For many years, the English‐speaking scientific community tended to regard *Mutual Aid* as the product of a scientific amateur who dabbled in evolutionary theory to justify his political project. However, this was more a reflection of the ignorance of that community than of Kropotkin's dilettantism (Gould 1988). As a young man, he had pursued a stellar scientific career, leading numerous geographical survey expeditions in western Russia and Scandinavia (Read 1942). Moreover, his response to Darwin's theory was not something that he personally developed, but reflected a longstanding (but untranslated) intellectual current in the Russian natural scientific community (Todes 1987). While Darwin had developed his theory in the context of an industrial revolution characterised by urbanisation, population density, capitalist competition, and growing economic inequalities, Russia was relatively economically underdeveloped and underpopulated. Moreover, much of its vast space was inhospitable, where the struggle for survival pitted the organism against its environment in the manner of Darwin's plant on the edge of a desert.

A common perception among Russian biologists was that Darwin's metaphor of the ‘struggle for existence’ required qualification. They argued that, despite his recognition of the different modes of struggle, Darwin was over‐reliant on Malthus, leading him to exaggerate the importance of intraspecific conflict. Instead, they contended that ‘the central aspect of the struggle for existence is the organism's struggle with abiotic conditions; organisms join forces to wage this struggle more effectively, and such mutual aid is favored by natural selection; since cooperation, not competition, dominates intraspecific relations’ (Todes 1987, p.545). These views were systematised by the zoologist, K. F. Kessler, whose evolutionary ‘law of mutual aid’, which held that ‘the more the individuals keep together, the more they mutually support each other, and the more are the chances of the species for surviving’ was cited by Kropotkin (1902, p.7) as the essence of his evolutionary theory.

### 2.3 Mutual Aid Versus Social Darwinism

Kropotkin's application of mutual aid to the evolution of human societies stood in contradiction to ‘social’ Darwinism, which purported to apply Darwin's theory of natural selection to human societies, asserting that social evolution entails competitive struggle between groups and individuals, leading to the survival of the fittest (Spencer 1864). In sharp contrast to this position, Kropotkin underlined ‘the overwhelming importance which sociable habits play in Nature and in the progressive evolution of… human beings’ (1902, p. xvi). These sociable habits in human beings provide ‘the possibility of working out those institutions which have enabled mankind to survive its hard struggle against Nature, and to progress, notwithstanding all the vicissitudes of its history’ (Kropotkin 1902, p. xvi).

Darwin was also opposed to social Darwinism, but for different reasons. In *The Descent of Man* (1896), he argued that social impulses are distinct from, and act in a contrary direction to, natural evolutionary processes. This position was further developed by T. H. Huxley, who characterised humans, because of their evolutionary position, as being subjected to two opposing forces. The first was the evolutionary struggle for existence, which involved a non‐moral competition for limited resources, in which only the fittest survived. The other was humanity's attempt to escape from this Hobbesian nightmare by means of ‘civilised’ society, which, being animated by morality, attempted to substitute ‘the state of mutual peace for that of mutual war’ (Huxley 1948, p.64). Kropotkin took objection to Huxley's argument as ‘a very incorrect representation of the facts of nature’ (1902, p. xiv) on the grounds that, in common with other animals, the natural (as well as the social) human impulse is more powerfully oriented to cooperation and support than to the elimination of the weak. Roughly schematised, the different perspectives on the nature/society duality can be described as conflict/cooperation for Darwinism; conflict/conflict for social Darwinism; and cooperation/cooperation for mutual aid.

### 2.4 The Politics of Mutual Aid

Kropotkin's study of ‘primitive’ societies led him to believe that people have always been sociable beings. He pointed to ‘the number and importance of mutual‐aid institutions which were developed… during the earliest clan‐period of mankind’ (1902, p. xv). He saw mutual aid reaching its peak in medieval European city states. But then, in the sixteenth century, ‘these centres of civilisation … were seized with senile debility’ (1902, p.215). In their place rose powerful militarised states which

systematically weeded out all institutions in which the mutual‐aid tendency had formerly found its expression … It was taught … that the institutions in which men formerly used to embody their needs of mutual support could not be tolerated in a properly organized State; that the State alone could “represent the bonds of union between its subjects.

(1902, p.226)

All of these pressures had a debilitating effect: ‘In proportion as the obligations towards the State grew in numbers the citizens were evidently relieved from their obligations towards each other’ (1902, p.226). Yet, Kropotkin argued that the impulse for mutual aid has not and cannot be extinguished, because it is a core part of our evolutionary being:

In our mutual relations every one of us has his moments of revolt against the fashionable individualistic creed of the day, and actions in which men are guided by their mutual aid inclinations constitute so great a part of our daily intercourse that if a stop to such actions could be put all further ethical progress would be stopped at once. Human society itself could not be maintained for even so much as the lifetime of one single generation.

(Kropotkin 1902, p.229)

All of this points to the need to undermine the state by engaging in mutual aid by whatever means possible – he supplied examples that ranged from solidarity strikes to socialist agitation; from cycling clubs to lifeboat associations – in short he advocated (without explicitly using the term) the development of anarchist social organisation in all spheres of life. Because he saw the prime culprit for the destruction of cooperative social structures as the coercive state, Kropotkin concentrated more on governmentality than economic relations in *Mutual Aid*, though elsewhere (e.g. Kropotkin 1906), he promotes a robust anarcho‐communist programme.

### 2.5 Kropotkin's American Reputation

It is important to note that Kropotkin was not writing in obscurity. At the time when Nutting and Dock published *A History*, he was seen as the leading figure of the powerful international anarchist movement. Kropotkin was revered by his comrades and vilified by his enemies. The Russian anarchist émigré (and trained nurse), Emma Goldman is a good example of the former: ‘We saw in him the father of modern anarchism, its revolutionary spokesman and brilliant exponent of its relation to science, philosophy, and progressive thought’ (Goldman 1931, p.509). Such admiration was matched by vilification. So, for example, a conspiracy theory was spread that Kropotkin was one of the leading plotters of the assassination of President McKinley in 1901 by a self‐proclaimed anarchist (Avrich 1980).

One of the governmental responses to the assassination of McKinley was the enactment of the Immigration Act in 1903. This was the first time that immigration legislation was used to exclude people from the USA on the basis of their political beliefs. Specifically and exclusively targeting anarchists, the law stated that ‘no person who disbelieves in or who is opposed to all organized government… shall be permitted to enter the United States’ (Immigration Act 1903, Sec. 38). The Act demonstrated the fear and opprobrium felt by US political, economic and social elites towards what they saw as the most potent challenge to the political and economic order they espoused. Kropotkin was henceforth a *persona non grata* in the USA.

Just 4 years after the Immigration Act, Nutting and Dock had the temerity to proclaim that the impulse underpinning nursing had been identified by such an undesirable alien. They could have easily rendered their position more acceptable by citing Kessler's law of mutual aid as their source, for example, but instead, they boldly nailed their colours to the mast. Given their positions in the pantheon of nursing leadership, this was remarkably brave.

## 3 Nursing as Mutual Aid

We have already noted Nutting and Dock's assertion of the fundamental evolutionary importance of the social instinct for ‘the protection of the younger and weaker of the herd by the older and stronger … [that] causes flocks to share the distress or attempt the rescue of the individual in peril’ (Nutting and Dock 1907a, p.5). To substantiate their position, they explain how Kropotkin's theory was based on scientific observation: ‘In all these scenes of animal life which passed before my eyes … I saw Mutual Aid and Mutual Support carried on to an extent which made me suspect in it a feature of the greatest importance for the maintenance of life, the preservation of each species, and its further evolution’ (Kropotkin 1902, p. ix, quoted by Nutting and Dock 1907a, pp.5‐6). They then recount how Kropotkin came to realise that his empirical findings were closely aligned with Kessler's theoretical explanation, namely the idea that ‘beside[s] the law of Mutual Struggle there is in Nature the law of Mutual Aid, which… for the progressive evolution of the species is far more important than the law of mutual contest’ (Kropotkin 1902 p.x, quoted by Nutting and Dock 1907a, p.6).

On this basis, they go on to attack Social Darwinian assumptions about the evolutionary struggle that involves the survival of the fittest in human societies, quoting Kropotkin's dismissal of these theories on ‘the relations between Darwinism and sociology’ (Kropotkin 1902, p. ix): ‘Kropotkin's observations convinced him that those animal tribes whose members assist each other the most have survived the most extensively and are the most capable of survival, and the theory that competition is the predominating law of life, and the “struggle for the means of existence… of every man against all other men, ‘a law of Nature,’… lacked confirmation from direct observation”’ (Nutting and Dock p. 6, Kropotkin, pp. ix–x). They follow this up with empirical examples that he gives of mutual aid being the predominant mode of interaction in ‘primitive societies’.

Connecting the theory of mutual aid with the position of nursing in society, Nutting and Dock start by citing Kropotkin's critique of the historiographical bias towards recording dramatic conflict: ‘“Historians and annalists,” says Kropotkin, “have chronicled wars and calamities, but have paid no attention to the life of the masses or the countless acts of mutual support and devotion”’ (1907a, p.7)^1^. For Nutting and Dock, nursing is the epitome of this historiographically hidden but socially ubiquitous impulse towards mutual support: ‘In attempting to study the history of nursing, which must always have existed in some form, however rude, we find long ages of silence on the subject, doubtless because of this tendency of historians to overlook what was usual and homely. During centuries of the time when some sort of chronicles recorded human progress, there is no mention of nursing as differentiated from empiric medicine’ (1907a, p.7). The core aim of their history was to fill in this biassed lacuna in order to demonstrate that nursing was a crucial instantiation of the core evolutionary impulse towards mutual aid.

### 3.1 The Ability to Engage in Mutual Aid

Just as Kropotkin chronicled the ebb and flow of the influence of mutual aid over time, Nutting and Dock took a socio‐historical view that saw the nurse's ‘calling’ as closely ‘linked with the general conditions of education and of liberty that obtain – as they rise, she rises, and as they sink, she falls’ (1907a, p.v). Their use of the female pronoun indicates that they were referring specifically to women's contingent access to knowledge and freedom. For Nutting and Dock, patriarchal control was anathema to the possibility of nursing as mutual aid. They made their point forcefully in their analysis of the ‘dark period’ of nursing between the seventeenth and nineteenth centuries when ‘the condition of the nursing art, the wellbeing of the patient, and the status of the nurse all sank to an indescribable level of degradation’ (1907a, p.499):

The state of degeneration to which men reduced the art of nursing during this time of their unrestricted rule, the general contempt to which they brought the nurse, the misery which the patient thereby suffered, bring a scathing indictment against the ofttime reiterated assertion of man's superior effectiveness, and teach in every branch of administration a lesson that, for the sake of the poor, the weak, and the suffering members of society ought never to be forgotten.

(1907a, p.501)

The implicit assumption in this argument (and throughout *A History*) is that the ability to engage in mutual aid is a function of the capacity to care, which, in turn, is more highly developed in women than in men. For Nutting and Dock, the female advantage in caring traits is due largely to the evolutionary extension of maternal instincts towards offspring to a more general disposition of caring. Their assumption that males and females were evolutionarily endowed with different comparative strengths in specific traits was in line with the evolutionary feminism of their day, which, while accepting Darwin's theory of sexual selection, rejected his conclusion that this led to male superiority. So, for example, Antoinette Blackwell, using data compiled by Darwin, constructed tables comparing the relative strengths of secondary sexual characteristics of the different sexes in numerous species, including humans. When all the relative strengths and weaknesses of the listed characteristics were added up, she contended that the result in every species was a balanced equation between males and females, which demonstrated ‘organic equilibrium in physiological and psychological equivalence of the sexes’ (Blackwell 1875, p.58).

### 3.2 The Debate about Means and Ends

The evolutionary theory of mutual aid and the political theory of anarchism are two distinct bodies of thought. While the former provides a theoretical basis for the latter, its acceptance does not necessarily entail acceptance of anarchism (Skyer et al. 2025). Like Kropotkin himself in *Mutual Aid*, Nutting and Dock did not make any explicit reference to anarchism in *A History*. Their primary problematic was not general political organisation but the social and historical role of nursing. While their bold accreditation of Kropotkin as their source indicates that they were far from unsympathetic to his political aspirations, there is evidence that the means they advocated to get to those ends differed from those favoured by their anarchist contemporaries.

The contrast was clearly illustrated by the conversations that occurred at the Henry Street Nurses' Settlement where Lavinia Dock lived and worked for so many years. In addition to meeting Kropotkin there, she came to know fellow nurse Emma Goldman. It was not coincidental that such anarchist luminaries should have contributed to the discursive milieu of the Settlement, which was far more than a public health hub. As part of its remit to develop a wide range of cultural and educational activities, it was a fecund forum for the discussion of social and political ideas. Through her friendship with a member of the Settlement, Goldman became part of that milieu:

Emma Lee was employed in the Nurses’ Settlement on Henry Street and I often visited her there, sometimes as the guest of the women at the head of the institution. Miss Lillian D. Wald, Lavinia Dock, and Miss MacDowell were among the first American women I met who felt an interest in the economic condition of the masses. They were genuinely concerned with the people of the East Side. My contact with them… brought me close to new American types, men and women of ideals, capable of fine, generous deeds. Like some of the Russian revolutionists they, too, had come from wealthy homes and had completely consecrated themselves to what they considered a great cause.

(Goldman 1931, p.160)

High praise indeed. However, she goes on to qualify that praise:

Yet their work seemed palliative to me. “Teaching the poor to eat with a fork is all very well,” I once said to Emma Lee; “but what good does it do if they have not the food? Let them first become the masters of life; they will then know how to eat and how to live.” She agreed with my view that, sincere as the settlement workers were, they were doing more harm than good.

(Goldman 1931, p.160)

If Goldman thought that the nursing leadership of Henry Street lacked sufficient radicalism, they in their turn felt that Goldman and her comrades lacked realism. As the founder of the settlement, Lillian Wald put it:

In some quarters mere radicalism has become perilously popular. The spirit of adventure, a kind of generous devotion not always balanced with knowledge of definite issues or the constructive processes that are under way, deflect forces that might be employed for immediate advances in social welfare.

(Wald 1915, p.277)

Thus Wald and Goldman epitomise the two competing strands of reform and revolution (Connelly 2010), embodying the tension between the nurse's obligation to ameliorate individual suffering at the point of need and the radical's imperative to eradicate the conditions that cause suffering. Lillian Wald, a close friend of Lavinia Dock, was involved in the creation of a dazzling number of progressive and reformist organisations, including the National Organisation for Public Health Nursing and the National Association for the Advancement of Colored People. All these organisations had the express purpose of reforming the state and society to promote social and economic equality (Cook 1979; Coss 1989; Connolly 2010).

Emma Goldman, in contrast, had no faith in the effectiveness of such reformist efforts. While accepting the need to make intermediate demands on issues such as wages and working hours, she was convinced that capitalism was so enslaving and dehumanising that it had to be overthrown. Similarly, she saw the state as a tool of oppression. For her, liberal democracy was a chimera and the right to vote merely an illusion of political influence. As a consequence, while she supported women's equality with men, she believed that it was naive to think that women's suffrage would lead to an improvement of the body politic. For her, the best strategy to attain freedom was direct action, including the use of political violence where necessary (Goldman 1931; Connolly 2010).

While there may seem to be a considerable gulf between Wald and Goldman, their assessments of each other are notable for their mutual respect. The role of the Henry Street Settlement as a forum for the exchange of progressive ideas likely facilitated comradely civility based on a shared progressive outlook:

As different as their political visions and choice of strategies were … Wald … and Goldman were dedicated to a future society that guaranteed economic security and the full development of individual potential for women and men on the basis of absolute equality.

(Cook, 1979, p.415)

## 4 Contemporary Dilemmas

### 4.1 The Debate about Means and Ends

If the early 20th century debate was about both means and ends, the 21st century nursing debate about anarchism is more about ends. While there seems to be agreement on the importance of bottom‐up, micro‐political action, there are different perspectives on the purpose of this sort of engagement. Martin and Laurin advocate micro‐political insurrection as an alternative to what they see as the unviable project of overturning of the state. They defend their ameliorative approach by arguing that ‘even if institutional power remains intact, individuals can embody anarchist philosophy by applying its’ [sic] precepts at home, school, and the workplace. Consequently, anarchism is not only about dreaming up future possibilities for sociopolitical engagement, but rather focusing primarily on the possibilities of changing the conditions in the *here and now*. In this sense, it is not a strictly utopian endeavor' (2023, p.5).

Oppedisano and Dillard‐Wright concur with Martin and Laurin's advocacy of micro‐insurgency as ‘a small but effective way that nurses can subvert the systems we work under to prioritise our patients over the rules and hierarchies of our institutions’ (Oppedisano and Dillard‐Wright 2024, p.5) but, for them, these subversions are not simply ends in themselves. Rather, they envisage them as prefigurative praxis designed to open up possibilities for large‐scale social changes. In tandem with collective direct action, they are'techniques to short‐circuit the wiring of pastoral power and governmentality' (2024, p.4). Elsewhere, Dillard‐Wright is even more explicit about the utopian aspiration to unmake exploitative structures: ‘Because the Capitalocene was relationally made, the product of the extraction orientation dictated by capitalism, it can likewise be “relationally unmade” in an effort to achieve something more sustainable, more human, more connected’ (Walsh et al. 2023, p.7).

This is not to say that Martin and Laurin confine anarchist praxis to individual actions; they also observe that ‘nurses can collectively respond to capitalist and neoliberal forces’ (2023, p.6). The examples they give include women's health services set up on feminist principles, clinics that are accessible to members of vulnerable populations, political protest against racist brutality, and industrial action for better terms and conditions. While these are all important radical strategies, they remain within the parameters of bourgeois social democracy (though perhaps outside the bounds permitted by bourgeois proto‐fascism).

It strikes me that this debate is taking place on similar terrain to that which occurred in early twentieth‐century nursing between radical reformists like Lavinia Dock and Lillian Wald and revolutionaries like Emma Goldman. However, it is not my purpose to judge the relative merits of ameliorative reform and utopian revolution beyond commenting that the rapid escalation of economic inequality and ecological precarity has significantly raised the stakes of the debate for contemporary nurses.

### 4.2 The Ability to Engage in Mutual Aid

A related dilemma concerns the very possibility of nurses engaging in mutual aid. Martin and Laurin are sanguine about this issue, arguing that mutual aid ‘is already at work in nursing' (2023, p.5). They provide a number of practical examples of its operationalisation, such as mutual help with difficult techniques, refusing to engage in language that assumes medical superordination, and maintaining professional solidarity with colleagues.

Dillard‐Wright is less optimistic: ‘As presently fashioned as part of the healthcare system, nursing *is not and cannot be* mutual aid (2022, p.6). Her critique of nursing echoes Spade's (2020) accusation that state‐sponsored non‐state institutions like charities and non‐profit organisations legitimise unjust systems by usurping control over people's lives and putting that control in the hands of a small groups of paid professionals. For Dillard‐Wright, nurses play a similar role in de‐legitimising people's lived experiences: ‘The simple act of defining people as patients, even, imposes hierarchy and defines their subjectivity in specific terms, fixing them in the healthcare gaze and creating docile bodies’ (2022, p.6). In this context, we might ask whether the injunction that ‘nurses should be aware of the importance of not criticising another nurse's work in front of patients’ (Martin and Laurin 2023, p.6) is an example of collegiate solidarity or an occupational strategy to set nursing ‘apart from communities as professionals, as experts, as authorities’ (Dillard‐Wright 2022 p.6).

Dillard‐Wright's Foucauldian critique of the nursing stance ‘where nurses know best and centre our expertize in a benevolent saviour fashion over the lived experiences of the people we accompany in care’ (2022, p.6) can be applied to Nutting and Dock's assumption that good care is a function of feminine beneficence. For mutual aid to be meaningful, it cannot rely solely upon nurses, but (as its title implies) must embody mutual acceptance and possession of autonomy and agency for all those involved in relationships of care.

However, at the other end of the spectrum, it is hard to avoid the conclusion that, if we take Foucault to his post‐structuralist limits and accept that ‘pastoral power’ ineluctably ensnares its subjects ‘in continuous networks of obedience’ and subjectifies them ‘through the compulsory extraction of truth’ (Foucault 2007, p.185), there is little or no possibility of developing a relationship of mutual aid between nurses and patients. Or can we plot a course between the naive denial of a nexus of power between professionals and the laity, and an indiscriminate cratology in which ‘power is everywhere’ (Foucault 1979, p.93) and where the answer to the question ‘Who fights against whom?’ is the desolate Hobbesian response ‘We all fight against each other’ (Foucault 1980, p.208)?

One possible route out of this dilemma involves the recognition that human agency is central to the dynamics of pastoral power (Martin and Waring 2018) in that social relations involve volitional agents rather than passive subjects of discursive practices (Caldwell 2007). This allows for the possibility of agents constituting their relationships in ways that are conducive to mutual aid. However, the radical equalising of professional/client relationships that this would involve indicates that, notwithstanding its conceptual distinctiveness, effective mutual aid requires anarchist political praxis. As Essex notes, at the core of anarchism is a ‘deep and generalized skepticism toward all authority’. He qualifies this statement by noting that ‘anarchism is not the outright rejection of authority.’ Rather, it is ‘a sort of prima facie rejection, that demands those with authority justify themselves, particularly when it comes to the exercise of power over others’ (2026, p.3). This scepticism towards the ‘theoretical authority’ (Essex 2026) of professionals finds its echoes in the increasing influence of person‐centred care as a framework for nursing practice (McCance and McCormack 2025). It is gratifying to observe that nursing is beginning to catch up (at least rhetorically) with anarchism's longstanding distrust of the authority of experts.

This is not to say that the anarchist approach to theoretical authority is a panacea. A significant problem arises from its over‐reliance on rational choice. Thus, another great Russian anarchist, Mikhail Bakunin, characterised his attitude to the recommendations of specialists as follows: ‘their authority is imposed upon me by no one, neither by men nor by God … I bow before the authority of special men because it is imposed upon me by my own reason’ (Bakunin 1920, p.36). This formal freedom to decide whether or not to accept professional knowledge and authority, while necessary, is not sufficient, given what is at stake for patients in healthcare encounters. Even if we do not go as far as Foucault in his assertion that power ‘forms knowledge, produces discourse’ (2000, p.120), it would be naïve to dismiss the complex nexus between knowledge and power that is embedded in the professional/client relationship (Cheek and Porter 1997). Formal freedom of choice based on rational decision making cannot account for what Habermas (1984) terms systematically distorted behaviour, which results from the manipulations involved in the maintenance of hierarchical and exploitive relations.

As Dillard‐Wright (2022) noted, the possibility of patients and nurses constructing egalitarian relationships at an interpersonal level is severely constrained by the hierarchical control of health service organisations as presently fashioned. Nursing anarchist commentators recognise the interlocking of the ‘theoretical authority’ of professionals and the ‘managerial authority’ (Essex 2026) of those who own and control healthcare institutions. In response, they tend to advocate the development of organically developed and community embedded alternatives of sufficiently modest scope to facilitate deliberative democratic participation (vide Martin and Laurin 2023; Malfait 2025; Essex 2026). While these prefigurative examples are exciting and life affirming, it is not entirely clear how they can address the problem of scale. Assuming that the complexities of 21st century healthcare will continue to require efficiencies of scale and manifold divisions of labour and expertise, then the ability to engage meaningfully in mutual aid will depend upon the development of forms of governance of large and complex healthcare systems based on autonomy, solidarity and responsibility (*vide* Parker et al. 2014). A tall order perhaps, but a problematic worth addressing in future anarchist nursing discussions.

## 5 Speculative Comparisons

To conclude discussion of Nutting and Dock's approach to mutual aid, I am going to speculatively compare it with two areas of contemporary thought which speak to their central concerns about the place of women in particular and humans in general, namely feminist and ecological theories.

### 5.1 The Comparison with Feminist Theory

Nutting and Dock's feminism was a product of its times. While Darwinian theories powerfully challenged the ‘spare rib’ and ‘curse of Eve’ origin myths, which provided biblical rationalisation for female oppression, ironically, Darwin's theory of sexual selection (1896) reinforced the Judaeo‐Christian doctrine of complementarianism, whereby he substituted divine assignation of different and complementary roles and responsibilities to men and women with evolutionary assignation. As already noted, the response of feminists of this era was not to challenge the essentialism involved, but to argue that the characteristics assigned to women were just as important as those attributed to men (Blackwell 1875). It was on this essentialising terrain that Nutting and Dock feminised the core values of mutual aid. But it also has to be said that complementarianism led to them (albeit sometimes begrudgingly) to accept male/medical authority over the key activities of diagnosis and prescription.

Nonetheless, they can be seen as precursors to feminists such as Carol Gilligan (1982) who identified a feminist ethics of care that emphasises the need for responsiveness in relationships and the importance of connection with others. Nutting and Dock and Gilligan took the same steps in arguing, first, that women have a ‘different voice’ in moral reasoning, and second, that the female voice is as valid as the male voice. Where they differ is that Gilligan did not speculate on the origins of difference. Indeed, over time she has become increasingly wary of essentialising gendered attributes, arguing that ‘within a patriarchal framework, the ethics of care is a “feminine” ethic, whereas within a democratic framework it is a human ethic’ (Gilligan 2011). This has led her to conclude that ‘the association of care and caring with women’ is an injustice inherent in patriarchy. It is difficult to align this cultural feminist position with Nutting and Dock's identification of women with caring on evolutionary grounds.

However, while cultural approaches to gender issues remain highly influential within feminist thought, the emergence of ‘new materialist’ feminism which reasserts the active role of nature (Barad 2003) has re‐opened the space for neo‐Darwinian feminism, which sees sexual difference as neither an essential attribute nor a cultural epiphenomenon, but as an evolutionary trait (Grosz 2005). If nothing else, these theoretical developments indicate that Nutting and Dock's attempts to forge an understanding of the problematic relationships between nature and culture, and sex and gender using evolutionary theory is not merely an historical anomaly.

### 5.2 The Comparison with Ecological Theory

By this stage in history, it has become painfully obvious that human hubris has led to the brink of planetary catastrophe, such that an ethic of human humility is desperately needed (Sadler‐Smith and Akstinaite 2022). This humility in turn mandates a posthumanist approach to our understanding of the world, in the sense that we need to appreciate that there is nothing exceptional about homo sapiens – it exists within a co‐dependent web of other species and inanimate systems (Cudworth and Hobden 2018).

Given their belief in the hierarchical superordination of contemporaneous humanity over both the ‘lower animals’ (1907a, p.9) and ‘primitive man’ (p. 12)^2^ Nutting and Dock were far from posthumanists, but that does not mean that their approach to the nature/culture relationship has nothing to contribute to current debates. First, as Darwinian evolutionists, they were wedded to a theory that binds humanity to the rest of the living world. Second, they recognised that part of this evolutionary chain was the impulse to mutual aid that was evident in many different species and all epochs of humanity.

This focus on the evolutionary basis of an ethic of care is something that tends to be underplayed in contemporary anarchist discourse. Thus, Spade, while arguing that mutual aid works ‘to meet survival needs’ (2020, p.14), resiles from evolutionary analysis, concentrating instead on political arguments about the capacity of mutual aid projects to mobilise opposition to social inequalities and injustices, along with social psychological arguments which posit that the solidarity and participatory problem‐solving inherent in mutual aid are social goods in their own right. The distancing of discursive practices from their interactivity with natural processes risks returning anarchism to a human‐centric view of the world (Cudworth and Hobden 2018). In contrast, an emphasis on mutual aid as an evolutionary dynamic could provide a solid basis for posthumanist approaches to nursing that recognise the symbiotic dependencies upon which health depends, and oppose the forces of extractive capitalism, neo‐colonialism and imperialism that are hell‐bent on destroying the health of the planet (Adam et al. 2021; Petrovskaya 2023).

## 6 Conclusion

It is always risky to read across to our own time from the perspectives of those who were engaged with different problems in a different era. This is certainly the case here. An argument could be made that many of Nutting and Dock's ideas are anachronistic. They were, albeit grudgingly, acquiescent to a complementarian patriarchy that placed men/doctors as superordinate decision‐makers; they paid scant regard to patients as volitional agents; and they took a parochial pride in the superiority of contemporaneous Anglo‐American nursing and a corresponding colonialist approach to non‐Western societies.

However, when looking at past ideas and comparing them to those of the present, it is possible to distinguish between contextual specificities and relatively enduring continuities (which can be both positive and negative). The great issues that Nutting and Dock grappled with, including the nature of caring, the relationship between feminism and nursing, and between nature and culture, are no less important or problematic now than they were 120 years ago. Indeed, their core aim to render visible the hitherto hidden contribution of nursing to health and wellbeing remains a work in progress, as the work of scholars like Davina Allen (2015) demonstrate. Moreover, their bravery in openly espousing the radical ideas of an opponent of the establishment is a lesson that contemporary nursing leaders might learn something from. But the most significant message we can take from them is that survival depends on mutual aid and that nurses have the potential to be key actors in mutual aid's instantiation. The fact that imminent ecological collapse has forced us to expand our focus from species to planetary survival only makes their message all the more pertinent.

## Funding

The author has nothing to report.

## Ethics Statement

The author has nothing to report.

## Conflicts of Interest

The author declares no conflicts of interest.

## Data Availability

The data that support the findings of this study are available in BRIAN at https://staffprofiles.bournemouth.ac.uk/display/porters#publications. These data were derived from the following resources available in the public domain: Bournemouth University, https://staffprofiles.bournemouth.ac.uk/display/porters#publications.

## Endnotes

1 It should be noted that this quotation does not display a level of fidelity to the original that modern scholarship would require. It is nonetheless faithful to Kropotkin's sentiments. Kropotkin's original text reads as follows: ‘The annalists of old never failed to chronicle the petty wars and calamities which harassed their contemporaries; but they paid no attention whatever to the life of the masses’ (p. 117). The rest of the quotation comes before, rather than after, this statement, where Kropotkin refers to ‘the cumbersome records which we prepare for the future historian’ which ‘hand down to posterity every kind of individual suffering; but they hardly bear any trace of the countless acts of mutual support and devotion which every one of us knows from his own experience’ (Kropotkin, 1902, p. 116).

2 The use of the term ‘primitive’, one that we have also seen used by Kropotkin, is an indication of the pervasiveness of colonialist ideology in white Europeans and those of white European descent, such that it infected even those who prided themselves on their radicalism. This attitude is most egregiously displayed in the final two volumes of A History of Nursing, which dealt with recent developments in nursing. So, for example, the chapter entitled ‘Nursing in new continents’ is largely a history of the development of settler colonial institutions in countries such as Australia, Canada and New Zealand. The racism involved becomes even more explicit when it addresses people subjected to occupation colonialism: ‘Africa is known as the “Dark Continent,” but darkness is giving place to dawn, and dawn with tropical rapidity to broad daylight. A powerful factor in this development is the trained nurse, who, following the [imperial] flag, has found her way to the heart of the continent’ (Dock 1912b, p. 222). To take just one example of the enlightening work of these flag‐following benefactors: ‘The hospital is maintained by the Universities ' Mission to Central Africa, and has a nursing staff of a Matron and five or six British certificated nurses, who take considerable pains to train the natives who work under them. The value of this work is great, as the African thus receives instruction in habits of order, method, and discipline, and in an appreciation of the value of time, which are foreign to him naturally’ (1912b, pp. 223‐224). The most dispiriting aspect of the deeply embedded racist ideology of nursing's foundational thinkers is that it continues to cast a pernicious shadow over our occupation (Kimani 2023).
